# 8-Bromo-1,3-diphenyl-2,3-dihydro-1*H*-naphtho­[1,2-*e*][1,3]oxazine

**DOI:** 10.1107/S1600536810026553

**Published:** 2010-07-17

**Authors:** Jerry P. Jasinski, Albert E. Pek, A. N. Mayekar, H. S. Yathirajan, B. Narayana

**Affiliations:** a Department of Chemistry, Keene State College, 229 Main Street, Keene, NH 03435-2001, USA; b Department of Studies in Chemistry, University of Mysore, Manasagangotri, Mysore 570 006, India, and SeQuent Scientific Ltd, Baikampady, New Mangalore 575 011 India; c Department of Studies in Chemistry, University of Mysore, Manasagangotri, Mysore 570 006, India; d Department of Studies in Chemistry, Mangalore University, Mangalagangotri 574 199, India

## Abstract

The title compound, C_24_H_18_BrNO, consists of an envelope-configured oxazine ring with a fused 8-bromo-1,3-diphenyl group and two bonded phenyl rings. The dihedral angles between the mean planes of the 8-bromo-1,3-diphenyl and the phenyl rings are 54.5 (6) and 87.4 (8)°, respectively. The oxazine is essentially coplanar with the 8-bromo-1,3-diphenyl [dihedral angle = 9.4 (1)°]. Weak C—H⋯π inter­actions contribute to the crystal packing.

## Related literature

For the anti­tumor activity of heterocycles containing oxazine, see: Benameur *et al.* (1996 ▶). For the treatment of Parkinson’s disease with naphthoxazines, see: Millan *et al.* (2004 ▶); Joyce *et al.* (2003 ▶). For the psychostimulating and anti­depressant activity of oxazines, see: Nozulak & Giger (1987 ▶). For their analgesic, anti­convulsant, anti­tubercular, anti­bacterial and anti­cancer activity, see: Kurz (2005 ▶); Turgut *et al.* (2007 ▶). For the range of their biological applications, see: Ohnacker & Scheffler (1960 ▶). For synthetic possibilities, see: Szatmari *et al.* (2003 ▶, 2004 ▶). For anti­cancer derivatives, see: Zhang & Li (2003 ▶). For related structures, see: Li *et al.* (2008 ▶); Sarojini *et al.* (2007 ▶); Şen *et al.* (2008 ▶); Yang *et al.* (2008 ▶); Zhang *et al.* (2009 ▶).
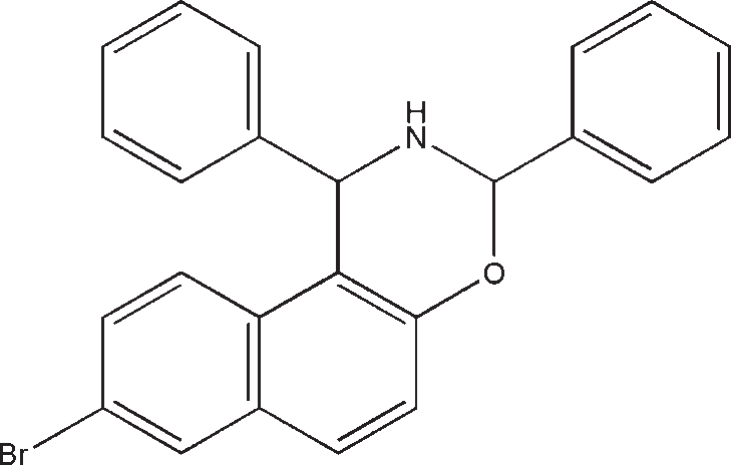


## Experimental

### 

#### Crystal data

C_24_H_18_BrNO
*M* *_r_* = 416.30
Monoclinic, 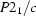

*a* = 7.7617 (11) Å
*b* = 20.092 (3) Å
*c* = 11.5094 (16) Å
β = 91.893 (2)°
*V* = 1793.9 (4) Å^3^
*Z* = 4
Mo *K*α radiation
μ = 2.31 mm^−1^
*T* = 100 K
0.55 × 0.50 × 0.35 mm

#### Data collection

Bruker APEXII CCD diffractometer
Absorption correction: multi-scan (*SADABS*; Bruker, 2008 ▶) *T*_min_ = 0.364, *T*_max_ = 0.499
14693 measured reflections
5341 independent reflections
4426 reflections with *I* > 2σ(*I*)
*R*_int_ = 0.025

#### Refinement

*R*[*F* ^2^ > 2σ(*F* ^2^)] = 0.040
*wR*(*F* ^2^) = 0.105
*S* = 1.03
5341 reflections
244 parameters
H-atom parameters constrained
Δρ_max_ = 1.64 e Å^−3^
Δρ_min_ = −0.84 e Å^−3^

### 

Data collection: *APEX2* (Bruker, 2008 ▶); cell refinement: *SAINT* (Bruker, 2008 ▶); data reduction: *SAINT*; program(s) used to solve structure: *SHELXS97* (Sheldrick, 2008 ▶); program(s) used to refine structure: *SHELXTL* (Sheldrick, 2008 ▶); molecular graphics: *SHELXTL*; software used to prepare material for publication: *SHELXTL* and *PLATON* (Spek, 2009 ▶).

## Supplementary Material

Crystal structure: contains datablocks global, I. DOI: 10.1107/S1600536810026553/tk2687sup1.cif

Structure factors: contains datablocks I. DOI: 10.1107/S1600536810026553/tk2687Isup2.hkl

Additional supplementary materials:  crystallographic information; 3D view; checkCIF report

## Figures and Tables

**Table 1:** C—H⋯π inter­actions (Å) *Cg*3, *Cg*4 and *Cg*5 are the centroids of the C4/C5/C7–C16, C13–C18 and C19–C24 rings, respectively.

*X*—H⋯*Cg*	*X*⋯*Cg*	H⋯*Cg*	H⋯Perp
C1–H1⋯*Cg*5^i^	3.357 (8)	2.80	2.67
C24–H18⋯*Cg*3^ii^	3.692 (9)	2.93	2.90
C22–H21⋯*Cg*4^iii^	3.547 (3)	2.68	2.61
C17–H24⋯*Cg*3^iv^	3.587 (8)	2.70	2.67

Symmetry codes: (i) −*x*, 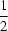 + *y*, 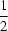 − *z*; (ii) −*x*, −*y*, 1 − *z*; (iii) −*x*, −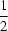 + *y*, 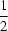 − *z*; (iv) −1 + *x*, *y*, *z*.

**Fig. 1.**
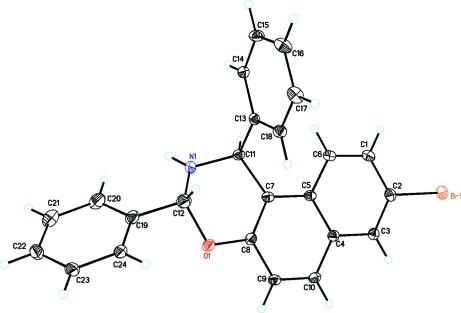
Molecular structure of (I) showing the atom labeling scheme and 40% probability displacement ellipsoids.

**Fig. 2.**
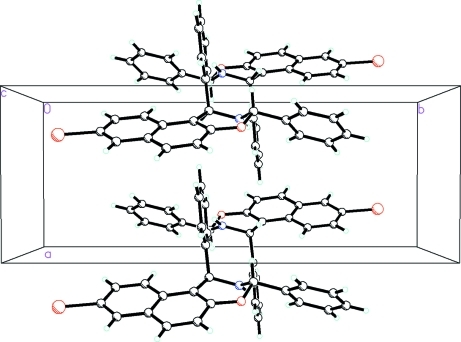
Packing diagram of (I) viewed down the c axis.

## supplementary crystallographic information

### Comment

Heterocycles containing the oxazine nucleus are found to possess a wide range of biological applications (Ohnacker & Scheffler *et al.*, 1960). 1,3-Oxazine heterocycles are of interest because they constitute an important class of natural and non-natural products. Many of them exhibit biological activity such as analgesic, anticonvulsant, antitubercular, antibacterial and anticancer (Kurz *et al.*, 2005; Turgut *et al.*, 2007). 1,3-Oxazine derivatives that display anticancer activity are also known as progesterone receptor agonists (Zhang *et al.*, 2003). Oxazine derivatives with a naphthalene ring, termed naphthoxazines, are used in the treatment of Parkinson's disease (Millan *et al.*, 2004; Joyce *et al.*, 2003). Naphthoxazines are also known for their psychostimulating and antidepressant activity (Nozulak & Giger *et al.*, 1987). Dihydrofuronaphth[1,3]oxazines have shown anti-tumor activity (Benameur *et al.*, 1996). In addition, naphthoxazines can be used as intermediates in the synthesis of *N*-substituted amino alcohols or in enantioselective synthesis of chiral amines. The tautomeric character of the 1,3-*O*,*N*-heterocycles offers a great number of synthetic possibilities (Szatmari *et al.*, 2003; Szatmari *et al.*, 2004). The crystal structures of a few naphthoxazines *viz*., 6-bromo-2,4-bis(3-methoxyphenyl)-3,4-dihydro-2*H*-1,3-naphthoxazine (Sarojini *et al.*, 2007), 3-(1,3 -benzodioxol-5-yl)-1-phenyl-2, 3-dihydro-1*H*-naphtho[1,2-*e*][1,3]oxazine (Yang *et al.*, 2008), 2-butyl-1,3-diphenyl-2,3-dihydro-1*H*-naphtho[1,2-*e*][1,3]oxazine (Li *et al.*, 2008), 1,3-di-3-pyridyl-2, 3-dihydro-1*H*-naphth-[1,2-*e*][1,3]oxazine (Şen *et al.*, 2008) and 2-benzyl-1,3-diphenyl-2,3-dihydro-1*H*-naphtho[1,2-*e*][1,3]oxazine (Zhang *et al.*, 2009) have been reported. In view of the importance of naphthoxazines, this paper reports the synthesis and crystal structure of the title compound, (I).

Compound (I) consists of an envelope configured oxazine (C8/C7/C11/N2/C12/O1) ring with a fused 8-bromo-1,3-diphenyl group and two bonded benzene rings (at C11 and C12) [puckering parameters Q, θ and φ = 0.460 (6) Å, 54.2 (7) °, and 259.144 (8) °, respectively] (Fig 1.); for an ideal envelope θ has a value of 54.7°. The dihedral angles between the mean planes of the 8-bromo-1,3-diphenyl (C1—C10) and the benzene rings (C13—C18 and C19—C24) are 54.5 (6) and 87.4 (8) °, respectively. The oxazine ring (C7/C8/O1/C12/N1) is essentially co-planar (dihedral angle = 9.4 (1)°) to the 8-bromo-1,3-diphenyl ring. Weak C–H···π interactions (Table 1) (Spek, 2003) are observed which contribute to crystal stability (Fig. 2).

### Experimental

Benzaldehyde (2.12 g, 0.02 mol) and 25–30% methanolic ammonia (10 ml) were added to 6-bromo-2-naphthol (2.23 g, 0.01 mol) in methanol (10 ml). The mixture was left to stand at ambient temperature for 3 days, during which the crystalline product separated out. The crude product was filtered off and washed with cold methanol. Crystals suitable for X-ray diffraction studies were grown by the slow evaporation of the acetonitrile solution (m.pt. 423–425 K).

### Refinement

All of the H atoms were placed in their calculated positions and then refined using the riding model with C—H = 0.93–0.98 Å, and with *U*_iso_(H) = 1.16–1.22*U*_eq_(C). The maximum and minimum residual electron density peaks of 1.64 and 0.84 eÅ^-3^, respectively, were located 1.01 Å and 0.06 Å from the C12 and H2 atoms, respectively.

### Crystal data

**Table:**

C_24_H_18_BrNO	*F*(000) = 848
*M**_r_* = 416.30	*D*_x_ = 1.541 Mg m^−^^3^
Monoclinic, *P*2_1_/*c*	Mo *K*α radiation, λ = 0.71073 Å
Hall symbol: -P 2ybc	Cell parameters from 5049 reflections
*a* = 7.7617 (11) Å	θ = 2.6–31.2°
*b* = 20.092 (3) Å	µ = 2.31 mm^−^^1^
*c* = 11.5094 (16) Å	*T* = 100 K
β = 91.893 (2)°	Block, colourless
*V* = 1793.9 (4) Å^3^	0.55 × 0.50 × 0.35 mm
*Z* = 4	

### Data collection

**Table:**

Bruker APEXII CCD diffractometer	5341 independent reflections
Radiation source: fine-focus sealed tube	4426 reflections with *I* > 2σ(*I*)
graphite	*R*_int_ = 0.025
ω scans	θ_max_ = 31.3°, θ_min_ = 2.0°
Absorption correction: multi-scan (*SADABS*; Bruker, 2008)	*h* = −11→10
*T*_min_ = 0.364, *T*_max_ = 0.499	*k* = −22→28
14693 measured reflections	*l* = −16→16

### Refinement

**Table:**

Refinement on *F*^2^	Primary atom site location: structure-invariant direct methods
Least-squares matrix: full	Secondary atom site location: difference Fourier map
*R*[*F*^2^ > 2σ(*F*^2^)] = 0.040	Hydrogen site location: inferred from neighbouring sites
*wR*(*F*^2^) = 0.105	H-atom parameters constrained
*S* = 1.03	*w* = 1/[σ^2^(*F*_o_^2^) + (0.0531*P*)^2^ + 1.779*P*] where *P* = (*F*_o_^2^ + 2*F*_c_^2^)/3
5341 reflections	(Δ/σ)_max_ = 0.001
244 parameters	Δρ_max_ = 1.64 e Å^−^^3^
0 restraints	Δρ_min_ = −0.83 e Å^−^^3^

### Special details

**Table:**

Geometry. All e.s.d.'s (except the e.s.d. in the dihedral angle between two l.s. planes) are estimated using the full covariance matrix. The cell e.s.d.'s are taken into account individually in the estimation of e.s.d.'s in distances, angles and torsion angles; correlations between e.s.d.'s in cell parameters are only used when they are defined by crystal symmetry. An approximate (isotropic) treatment of cell e.s.d.'s is used for estimating e.s.d.'s involving l.s. planes.
Refinement. Refinement of *F*^2^ against ALL reflections. The weighted *R*-factor *wR* and goodness of fit *S* are based on *F*^2^, conventional *R*-factors *R* are based on *F*, with *F* set to zero for negative *F*^2^. The threshold expression of *F*^2^ > σ(*F*^2^) is used only for calculating *R*-factors(gt) *etc*. and is not relevant to the choice of reflections for refinement. *R*-factors based on *F*^2^ are statistically about twice as large as those based on *F*, and *R*- factors based on ALL data will be even larger.

### Fractional atomic coordinates and isotropic or equivalent isotropic displacement parameters (Å^2^)

**Table:**

	*x*	*y*	*z*	*U*_iso_*/*U*_eq_	
Br1	0.26837 (3)	0.385264 (10)	0.377762 (19)	0.02407 (8)	
C11	0.1161 (2)	0.04955 (10)	0.22310 (16)	0.0149 (4)	
H16	0.1739	0.0708	0.1584	0.018*	
C7	0.1858 (2)	0.08276 (10)	0.33364 (16)	0.0137 (3)	
C5	0.2088 (2)	0.15294 (10)	0.34152 (16)	0.0144 (3)	
C8	0.2413 (3)	0.04374 (10)	0.42586 (16)	0.0149 (4)	
C4	0.2910 (2)	0.18121 (10)	0.44282 (16)	0.0153 (4)	
C9	0.3260 (3)	0.07139 (10)	0.52570 (17)	0.0172 (4)	
H9	0.3645	0.0437	0.5860	0.021*	
C10	0.3511 (3)	0.13830 (11)	0.53356 (17)	0.0177 (4)	
H10	0.4081	0.1561	0.5988	0.021*	
C3	0.3107 (3)	0.25095 (10)	0.45246 (17)	0.0169 (4)	
H3	0.3651	0.2693	0.5181	0.020*	
C2	0.2494 (3)	0.29135 (11)	0.36489 (18)	0.0185 (4)	
C1	0.1708 (3)	0.26470 (11)	0.26294 (18)	0.0193 (4)	
H1	0.1321	0.2928	0.2034	0.023*	
C6	0.1517 (3)	0.19715 (10)	0.25210 (17)	0.0170 (4)	
H6	0.1000	0.1799	0.1846	0.020*	
N1	0.1653 (3)	−0.02094 (9)	0.22166 (16)	0.0231 (4)	
H2	0.2104	−0.0405	0.1636	0.028*	
C12	0.1297 (3)	−0.05220 (11)	0.32674 (19)	0.0220 (4)	
H13	0.0061	−0.0483	0.3402	0.026*	
O1	0.2269 (2)	−0.02379 (7)	0.42750 (12)	0.0207 (3)	
C13	−0.0762 (2)	0.05797 (9)	0.19911 (16)	0.0131 (3)	
C14	−0.1404 (3)	0.05611 (10)	0.08416 (17)	0.0154 (4)	
H27	−0.0651	0.0515	0.0235	0.018*	
C19	0.1801 (3)	−0.12560 (11)	0.33218 (19)	0.0232 (4)	
C24	0.1387 (3)	−0.16039 (11)	0.43087 (18)	0.0215 (4)	
H18	0.0831	−0.1385	0.4901	0.026*	
C22	0.2638 (3)	−0.26054 (12)	0.3568 (2)	0.0261 (5)	
H21	0.2919	−0.3053	0.3654	0.031*	
C23	0.1781 (3)	−0.22716 (11)	0.44351 (19)	0.0233 (4)	
H22	0.1473	−0.2498	0.5102	0.028*	
C20	0.2633 (3)	−0.15863 (12)	0.2441 (2)	0.0279 (5)	
H19	0.2902	−0.1361	0.1765	0.033*	
C21	0.3069 (3)	−0.22641 (13)	0.2574 (2)	0.0284 (5)	
H20	0.3648	−0.2483	0.1991	0.034*	
C15	−0.3168 (3)	0.06123 (11)	0.06044 (18)	0.0196 (4)	
H26	−0.3592	0.0590	−0.0160	0.023*	
C18	−0.1906 (3)	0.06585 (10)	0.28794 (17)	0.0167 (4)	
H23	−0.1490	0.0669	0.3646	0.020*	
C17	−0.3669 (3)	0.07221 (11)	0.26411 (19)	0.0213 (4)	
H24	−0.4421	0.0781	0.3245	0.026*	
C16	−0.4300 (3)	0.06968 (11)	0.1503 (2)	0.0222 (4)	
H25	−0.5477	0.0736	0.1340	0.027*	

### Atomic displacement parameters (Å^2^)

**Table:**

	*U*^11^	*U*^22^	*U*^33^	*U*^12^	*U*^13^	*U*^23^
Br1	0.03207 (13)	0.01420 (11)	0.02574 (12)	0.00133 (8)	−0.00214 (8)	0.00038 (8)
C11	0.0146 (8)	0.0182 (9)	0.0117 (8)	0.0015 (7)	0.0003 (6)	−0.0008 (7)
C7	0.0106 (8)	0.0179 (9)	0.0127 (8)	−0.0001 (7)	0.0007 (6)	0.0006 (7)
C5	0.0114 (8)	0.0183 (9)	0.0136 (8)	0.0001 (7)	−0.0003 (6)	0.0010 (7)
C8	0.0179 (9)	0.0130 (9)	0.0138 (8)	−0.0031 (7)	0.0021 (7)	0.0006 (7)
C4	0.0147 (8)	0.0167 (9)	0.0144 (8)	0.0003 (7)	0.0011 (6)	−0.0002 (7)
C9	0.0229 (10)	0.0164 (9)	0.0122 (8)	−0.0002 (7)	−0.0018 (7)	0.0020 (7)
C10	0.0228 (10)	0.0171 (9)	0.0130 (8)	−0.0006 (7)	−0.0030 (7)	−0.0005 (7)
C3	0.0181 (9)	0.0148 (9)	0.0177 (9)	0.0003 (7)	−0.0002 (7)	−0.0014 (7)
C2	0.0196 (9)	0.0150 (9)	0.0209 (9)	0.0007 (7)	0.0017 (7)	0.0014 (7)
C1	0.0184 (9)	0.0198 (10)	0.0194 (9)	0.0017 (7)	−0.0024 (7)	0.0034 (8)
C6	0.0161 (9)	0.0190 (9)	0.0158 (8)	0.0005 (7)	−0.0018 (7)	0.0016 (7)
N1	0.0328 (10)	0.0190 (9)	0.0171 (8)	0.0099 (7)	−0.0040 (7)	−0.0050 (7)
C12	0.0231 (10)	0.0217 (10)	0.0210 (10)	−0.0012 (8)	−0.0007 (8)	−0.0004 (8)
O1	0.0352 (8)	0.0141 (7)	0.0127 (6)	−0.0047 (6)	−0.0025 (6)	0.0015 (5)
C13	0.0133 (8)	0.0110 (8)	0.0151 (8)	−0.0006 (6)	0.0003 (6)	0.0008 (6)
C14	0.0172 (9)	0.0139 (9)	0.0150 (8)	0.0003 (7)	−0.0006 (7)	−0.0001 (7)
C19	0.0342 (12)	0.0167 (10)	0.0184 (9)	−0.0058 (8)	−0.0035 (8)	−0.0003 (7)
C24	0.0310 (11)	0.0160 (10)	0.0172 (9)	−0.0010 (8)	−0.0044 (8)	−0.0007 (7)
C22	0.0271 (11)	0.0185 (10)	0.0322 (12)	0.0030 (8)	−0.0083 (9)	−0.0003 (9)
C23	0.0323 (11)	0.0170 (10)	0.0202 (9)	−0.0038 (8)	−0.0049 (8)	0.0040 (8)
C20	0.0375 (13)	0.0260 (12)	0.0202 (10)	−0.0105 (10)	0.0029 (9)	−0.0002 (9)
C21	0.0252 (11)	0.0316 (13)	0.0285 (11)	−0.0040 (9)	0.0024 (9)	−0.0091 (10)
C15	0.0167 (9)	0.0214 (10)	0.0202 (9)	−0.0025 (7)	−0.0060 (7)	0.0015 (8)
C18	0.0183 (9)	0.0178 (9)	0.0140 (8)	−0.0001 (7)	0.0015 (7)	0.0026 (7)
C17	0.0156 (9)	0.0228 (10)	0.0259 (10)	0.0000 (8)	0.0055 (8)	0.0060 (8)
C16	0.0125 (9)	0.0226 (10)	0.0313 (11)	−0.0033 (7)	−0.0018 (8)	0.0084 (9)

### Geometric parameters (Å, °)

**Table:**

Br1—C2	1.898 (2)	C12—C19	1.527 (3)
C11—N1	1.467 (3)	C12—H13	0.9800
C11—C13	1.518 (3)	C13—C18	1.385 (3)
C11—C7	1.520 (3)	C13—C14	1.399 (3)
C11—H16	0.9800	C14—C15	1.391 (3)
C7—C8	1.377 (3)	C14—H27	0.9300
C7—C5	1.424 (3)	C19—C24	1.381 (3)
C5—C6	1.420 (3)	C19—C20	1.388 (3)
C5—C4	1.428 (3)	C24—C23	1.383 (3)
C8—O1	1.361 (2)	C24—H18	0.9300
C8—C9	1.418 (3)	C22—C21	1.384 (4)
C4—C3	1.414 (3)	C22—C23	1.390 (3)
C4—C10	1.421 (3)	C22—H21	0.9300
C9—C10	1.361 (3)	C23—H22	0.9300
C9—H9	0.9300	C20—C21	1.410 (4)
C10—H10	0.9300	C20—H19	0.9300
C3—C2	1.367 (3)	C21—H20	0.9300
C3—H3	0.9300	C15—C16	1.389 (3)
C2—C1	1.410 (3)	C15—H26	0.9300
C1—C6	1.370 (3)	C18—C17	1.393 (3)
C1—H1	0.9300	C18—H23	0.9300
C6—H6	0.9300	C17—C16	1.384 (3)
N1—C12	1.399 (3)	C17—H24	0.9300
N1—H2	0.8600	C16—H25	0.9300
C12—O1	1.477 (3)		
			
N1—C11—C13	111.12 (16)	O1—C12—C19	102.55 (16)
N1—C11—C7	110.33 (15)	N1—C12—H13	108.8
C13—C11—C7	115.09 (16)	O1—C12—H13	108.8
N1—C11—H16	106.6	C19—C12—H13	108.8
C13—C11—H16	106.6	C8—O1—C12	114.47 (15)
C7—C11—H16	106.6	C18—C13—C14	118.95 (17)
C8—C7—C5	118.64 (17)	C18—C13—C11	121.92 (17)
C8—C7—C11	119.26 (18)	C14—C13—C11	119.11 (17)
C5—C7—C11	121.90 (16)	C15—C14—C13	120.09 (19)
C6—C5—C7	122.55 (17)	C15—C14—H27	120.0
C6—C5—C4	117.61 (18)	C13—C14—H27	120.0
C7—C5—C4	119.84 (17)	C24—C19—C20	119.0 (2)
O1—C8—C7	123.66 (17)	C24—C19—C12	117.2 (2)
O1—C8—C9	114.54 (17)	C20—C19—C12	123.8 (2)
C7—C8—C9	121.75 (18)	C19—C24—C23	121.4 (2)
C3—C4—C10	120.77 (18)	C19—C24—H18	119.3
C3—C4—C5	120.17 (18)	C23—C24—H18	119.3
C10—C4—C5	119.06 (18)	C21—C22—C23	119.2 (2)
C10—C9—C8	120.16 (18)	C21—C22—H21	120.4
C10—C9—H9	119.9	C23—C22—H21	120.4
C8—C9—H9	119.9	C24—C23—C22	120.1 (2)
C9—C10—C4	120.50 (18)	C24—C23—H22	119.9
C9—C10—H10	119.8	C22—C23—H22	119.9
C4—C10—H10	119.8	C19—C20—C21	119.9 (2)
C2—C3—C4	119.77 (18)	C19—C20—H19	120.1
C2—C3—H3	120.1	C21—C20—H19	120.1
C4—C3—H3	120.1	C22—C21—C20	120.3 (2)
C3—C2—C1	121.2 (2)	C22—C21—H20	119.8
C3—C2—Br1	120.56 (16)	C20—C21—H20	119.8
C1—C2—Br1	118.21 (15)	C16—C15—C14	120.37 (19)
C6—C1—C2	119.62 (19)	C16—C15—H26	119.8
C6—C1—H1	120.2	C14—C15—H26	119.8
C2—C1—H1	120.2	C13—C18—C17	120.99 (18)
C1—C6—C5	121.57 (18)	C13—C18—H23	119.5
C1—C6—H6	119.2	C17—C18—H23	119.5
C5—C6—H6	119.2	C16—C17—C18	119.8 (2)
C12—N1—C11	111.40 (17)	C16—C17—H24	120.1
C12—N1—H2	124.3	C18—C17—H24	120.1
C11—N1—H2	124.3	C17—C16—C15	119.76 (19)
N1—C12—O1	113.24 (18)	C17—C16—H25	120.1
N1—C12—C19	114.31 (19)	C15—C16—H25	120.1
			
N1—C11—C7—C8	−16.2 (2)	C7—C11—N1—C12	48.5 (2)
C13—C11—C7—C8	110.5 (2)	C11—N1—C12—O1	−62.2 (2)
N1—C11—C7—C5	158.55 (18)	C11—N1—C12—C19	−179.14 (17)
C13—C11—C7—C5	−74.7 (2)	C7—C8—O1—C12	−6.1 (3)
C8—C7—C5—C6	−178.32 (19)	C9—C8—O1—C12	176.55 (18)
C11—C7—C5—C6	6.9 (3)	N1—C12—O1—C8	39.9 (2)
C8—C7—C5—C4	1.2 (3)	C19—C12—O1—C8	163.56 (18)
C11—C7—C5—C4	−173.63 (17)	N1—C11—C13—C18	97.4 (2)
C5—C7—C8—O1	−179.57 (18)	C7—C11—C13—C18	−28.9 (3)
C11—C7—C8—O1	−4.6 (3)	N1—C11—C13—C14	−81.0 (2)
C5—C7—C8—C9	−2.4 (3)	C7—C11—C13—C14	152.64 (18)
C11—C7—C8—C9	172.48 (18)	C18—C13—C14—C15	−0.9 (3)
C6—C5—C4—C3	1.0 (3)	C11—C13—C14—C15	177.60 (19)
C7—C5—C4—C3	−178.53 (18)	N1—C12—C19—C24	−176.1 (2)
C6—C5—C4—C10	−179.44 (18)	O1—C12—C19—C24	60.9 (2)
C7—C5—C4—C10	1.1 (3)	N1—C12—C19—C20	3.5 (3)
O1—C8—C9—C10	178.84 (19)	O1—C12—C19—C20	−119.5 (2)
C7—C8—C9—C10	1.5 (3)	C20—C19—C24—C23	0.0 (3)
C8—C9—C10—C4	0.8 (3)	C12—C19—C24—C23	179.6 (2)
C3—C4—C10—C9	177.5 (2)	C19—C24—C23—C22	1.1 (3)
C5—C4—C10—C9	−2.1 (3)	C21—C22—C23—C24	−0.8 (3)
C10—C4—C3—C2	−179.0 (2)	C24—C19—C20—C21	−1.2 (3)
C5—C4—C3—C2	0.6 (3)	C12—C19—C20—C21	179.2 (2)
C4—C3—C2—C1	−1.9 (3)	C23—C22—C21—C20	−0.4 (3)
C4—C3—C2—Br1	178.18 (15)	C19—C20—C21—C22	1.4 (4)
C3—C2—C1—C6	1.5 (3)	C13—C14—C15—C16	1.4 (3)
Br1—C2—C1—C6	−178.55 (16)	C14—C13—C18—C17	−0.3 (3)
C2—C1—C6—C5	0.2 (3)	C11—C13—C18—C17	−178.75 (19)
C7—C5—C6—C1	178.12 (19)	C13—C18—C17—C16	1.0 (3)
C4—C5—C6—C1	−1.4 (3)	C18—C17—C16—C15	−0.4 (3)
C13—C11—N1—C12	−80.4 (2)	C14—C15—C16—C17	−0.8 (3)

### Table 1 C—H···π interactions (Å)

Cg3, Cg4 and Cg5 are the centroids of the C4/C5/C7–C16, C13–C18 and C19–C24 rings, respectively.

**Table:**

*X*—H···*Cg*	*X*···*Cg*	H···*Cg*	H···Perp
C1–H1···Cg5^i^	3.357 (8)	2.80	2.67
C24–H18···Cg3^ii^	3.692 (9)	2.93	2.90
C22–H21···Cg4^iii^	3.547 (3)	2.68	2.61
C17–H24···Cg3^iv^	3.587 (8)	2.70	2.67

Symmetry codes: (i) -x, 1/2+y, 1/2-z ; (ii) -x, -y, 1-z ; (iii) -x, -1/2+y, 1/2-z ; (iv) -1+x, y, z.
